# Single-cell transcriptomic and chromatin accessibility analyses of dairy cattle peripheral blood mononuclear cells and their responses to lipopolysaccharide

**DOI:** 10.1186/s12864-022-08562-0

**Published:** 2022-04-30

**Authors:** Yahui Gao, Jianbin Li, Gaozhan Cai, Yujiao Wang, Wenjing Yang, Yanqin Li, Xiuxin Zhao, Rongling Li, Yundong Gao, Wenbin Tuo, Ransom L. Baldwin, Cong-jun Li, Lingzhao Fang, George E. Liu

**Affiliations:** 1 Institute of Animal Science and Veterinary Medicine, Shandong Academy of Agricultural Sciences, No.202, Gongyebei Road, Jinan, 250100 China; 2 Animal Genomics and Improvement Laboratory, BARC, USDA-ARS, Beltsville, MD 20705 USA; 3 Shandong Ox Livestock Breeding Co., Ltd, Jinan, 250100 China; 4 College of Animal Science and Technology, China Agricultural University, Beijing, 100193 China; 5 Animal Parasitic Diseases Laboratory, BARC, USDA-ARS, Beltsville, MD 20705 USA; 6 MRC Human Genetics Unit at the Institute of Genetics and Molecular Medicine, University of Edinburgh, Edinburgh, EH4 2XU UK

**Keywords:** Cattle, Peripheral blood mononuclear cell, Lipopolysaccharide, Single-cell RNA-seq, Single-cell ATAC-seq

## Abstract

**Background:**

Gram-negative bacteria are important pathogens in cattle, causing severe infectious diseases, including mastitis. Lipopolysaccharides (LPS) are components of the outer membrane of Gram-negative bacteria and crucial mediators of chronic inflammation in cattle. LPS modulations of bovine immune responses have been studied before. However, the single-cell transcriptomic and chromatin accessibility analyses of bovine peripheral blood mononuclear cells (PBMCs) and their responses to LPS stimulation were never reported.

**Results:**

We performed single-cell RNA sequencing (scRNA-seq) and single-cell sequencing assay for transposase-accessible chromatin (scATAC-seq) in bovine PBMCs before and after LPS treatment and demonstrated that seven major cell types, which included CD4 T cells, CD8 T cells, and B cells, monocytes, natural killer cells, innate lymphoid cells, and dendritic cells. Bioinformatic analyses indicated that LPS could increase PBMC cell cycle progression, cellular differentiation, and chromatin accessibility. Gene analyses further showed significant changes in differential expression, transcription factor binding site, gene ontology, and regulatory interactions during the PBMC responses to LPS. Consistent with the findings of previous studies, LPS induced activation of monocytes and dendritic cells, likely through their upregulated TLR4 receptor. NF-κB was observed to be activated by LPS and an increased transcription of an array of pro-inflammatory cytokines, in agreement that NF-κB is an LPS-responsive regulator of innate immune responses. In addition, by integrating LPS-induced differentially expressed genes (DEGs) with large-scale GWAS of 45 complex traits in Holstein, we detected trait-relevant cell types. We found that selected DEGs were significantly associated with immune-relevant health, milk production, and body conformation traits.

**Conclusion:**

This study provided the first scRNAseq and scATAC-seq data for cattle PBMCs and their responses to the LPS stimulation to the best of our knowledge. These results should also serve as valuable resources for the future study of the bovine immune system and open the door for discoveries about immune cell roles in complex traits like mastitis at single-cell resolution.

**Supplementary Information:**

The online version contains supplementary material available at 10.1186/s12864-022-08562-0.

## Introduction

Mastitis is the most severe economic and health problem associated with dairy cow herds, affecting milk yield, milk composition, and productive life. Gram-negative bacteria are one of the important pathogens in cattle causing severe diseases, including mastitis and digestive tract infections. Lipopolysaccharides (LPS), also known as endotoxins, are components of the outer membrane of Gram-negative bacteria and crucial mediators of chronic inflammation in cattle suffering from clinical and subclinical infections caused by the bacteria. LPS exposure can result in elevated levels of local or systemic inflammation, which could compromise animal wellbeing and productivity [1, 2]. In mammals, the innate immune system serves as the first line of defense involving sensing pathogen-associated molecular patterns (PAMPs) and launching innate immune responses against the infections. LPS, a PAMP of the Gram-negative bacteria, is a highly potent activator of the innate immune system, eliciting strong inflammatory responses in infected animals [3]. The cells of the innate immune system, including monocytes (Mono), dendritic cells (DC), and granulocytes, function as the first line of defense upon encounter of infectious agents. Phagocytic macrophages, cytotoxic natural killer (NK) cells, and γδ T cells also play a crucial role in the innate immunity [4, 5]. Studies have been conducted to demonstrate the mechanisms by which LPS modulates the immune responses in vivo and in vitro. LPS can activate cellular responses by binding to the TLR4/CD14/MD2 receptor complex and activating pro-inflammatory transcription factors [6, 7]. Activated monocytes and DCs release nitric oxide, interleukin-1 (IL-1), IL-6, tumor necrosis factor-alpha (TNFα), and other factors [8]. Additionally, the innate immune cells such as monocytes and DC play a crucial role in bridging the innate and acquired immunities by responding to various PAMPs and serving as antigen-presenting cells (APCs) in the context of major histocompatibility complexes (MHC) [9]. The APCs must be adequately activated and conditioned upon their engagement with T cells, resulting in T cell activation in the presence of a cytokine and cell surface costimulatory molecule milieu, which is essential for the development of recall T cell responses required for host defense and protection. Surface marker genes on many immune cell types, like B cells and T cells, have been extensively studied [10]. For example, based on the expression levels of CD14 and CD16, monocytes can be divided into two types in the human blood [11].

A bulk human RNA-seq study demonstrated that LPS-responsive genes could be characterized as two co-regulated programs, i.e., the “antiviral-like” program and “inflammatory-like” program, based on their expression profiles [12]. The antiviral program is mainly mediated by interferon regulatory factors (IRFs). In contrast, the inflammatory program is primarily mediated by the Nuclear factor kappa-light-chain-enhancer of activated B cells (NF-κB) [12]. Single-cell-based analyses have been used to define human and mouse immune cells [13–15] and their responses to LPS. Additionally, single-cell RNA-seq studies further partitioned the inflammatory program genes into two modules, a peaked inflammatory module consisting of genes such as *TNF*, *IL1B*, and *CXCL2* that responded rapidly, yet transiently, when stimulated by LPS, and a sustained inflammatory module which included genes such as *Mmp14*, *Marco*, and *IL6,* exhibiting a continued rise in expression under LPS stimulation [13, 16].

The cell types and functions of cattle peripheral blood mononuclear cells (PBMCs) have been extensively studied [17–20]. In general, cattle PBMCs, similar to those of mammals, consist of primarily T and B cells, NK cells, monocytes, and DC [17, 20]. Cattle PBMC composition is unique in that young calves have higher levels of gamma/delta (γδ) T cell receptor (TCR) positive T cells in comparison to those of humans and mice [18]. However, large-scale single-cell analyses in cattle PBMCs have never been reported. There is a need to document the gene transcriptional, chromatin accessibility, and gene-based changes in PBMCs at the single-cell resolution before and after LPS stimulation. These studies will permit investigators to interrogate complex cellular regulations and interactions and delineate cell differentiation and lineage relationships within a sample of heterogeneous cell populations at the single-cell level. They will facilitate further understanding of LPS-mediated bovine PBMC responses and complement the existing methodologies determining PBMC cell types and functions. This is particularly important in cattle or other livestock species. There is a general lack of critical immunological reagents for thorough profiling of cell phenotypes, activation status, and cytokine production.

This study presents the first cattle single-cell PBMC profiling and their responses to LPS stimulation in vitro. The analyses of scRNA-seq data of the present study demonstrate robust clustering and assignment of naïve bovine PBMC populations and cell type-specific responses to LPS at the single-cell level. This study reports trait-relevant cell types and genes underlying complex traits by integrating LPS-induced DEGs with large-scale GWAS of 45 complex Holstein traits.

## Results

### Data generation and quality assessment

Using the 10 × Genomics Chromium Controller [21], we performed scRNA-seq and scATAC-seq of Holstein PBMC samples treated without (Control) or with LPS for 2 h (2 h-LPS), 4 h (4 h-LPS), and 8 h (8 h-LPS). We sequenced a total of 30,756 single cells with approximately 62,254 reads per cell (Table S1). After quality filtering and integration, we obtained 26,141 single cells, corresponding to a median of 4,581 unique molecular identifiers per cell and ~ 15,000 total genes in the whole population. Overall, we obtained 7,107 (Control), 9,174 (2 h-LPS), 6,741 (4 h-LPS), and 3,119 (8 h-LPS) cells.

### Cell clustering and cell type assignment

**Fig. 1:**
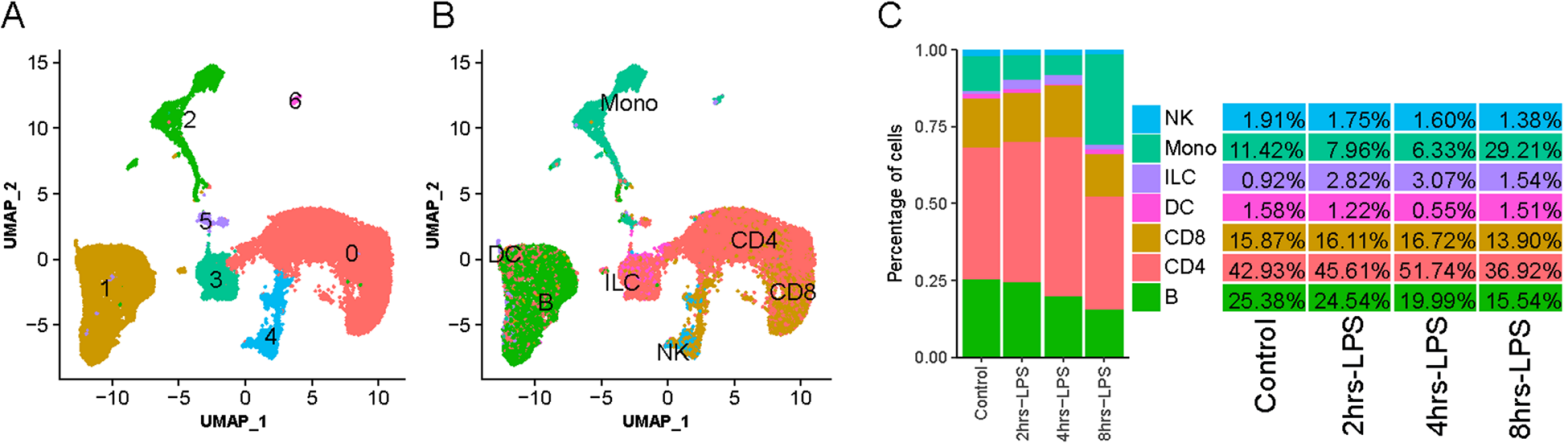
Cluster analysis of single-cell transcriptomes using four cattle PBMC samples. **A** UMAP projection plot showing seven major clusters of the 26,141 individual cell transcriptomes from all four PBMC samples. **B** The cell types were annotated using Azimuth (https://satijalab.org/azimuth/), based on their similarity to the human PBMC reference. **C** Plots and relative proportions of seven clusters/cell types across four PBMC samples, as annotated in **B**. The percentages in the table represent the relative proportions of cell types in four samples

Using Seurat v3.2 [22], we performed a graph-based clustering on cells according to the gene expression profiles. After visualizing the Uniform Manifold Approximation and Projection (UMAP) plots, we found that the single-cell transcriptomes of the four samples analyzed were similar (Figure S1A and 1B), indicating a high degree of reproducibility among them. We obtained a total of 7 distinct clusters designated by Cluster (C)0, C1, C2, C3, C4, C5, and C6 (Fig. 1A). We utilized canonical marker genes of immune cells derived from published literature and the online database PanglaoDB Field [23] to assign cell types. Based on gene expression patterns, we generated violin plots (Figure S2A) and UMAP projections (Figure S2B) for each gene. We then assigned immune cell types in cattle PBMC samples based on the combined unique patterns of these cell marker gene expressions as shown in parentheses (Figure S2A, Table S2). For example, CD4 T cells (*CD4*, *CD5*, and *LEF1*) and CD8 T cells (CD8A and CD8B) in C0, B cells (*MS4A1*, *CD79A*, *CD79B,* and VPREB3) in C1, monocytes (*CD14*, *S100A12*, *ADGRE1*, *MEFV*, and *HCK*) in C2 and C5, innate lymphoid cells (ILCs) (*SLC4A4*, *PLIN3*, and *COL5A1*) in C3, NK cells (*GNLY*, *NKG7*, *CTSW*, *PRF1*, and *IL2RB*) in C4, and DCs (*IRF8* and *CD83*) in C1 and C6. Of note, some combinations of these top marker genes were uniquely expressed in only one cell type, such as *CD14* and *S100A12* (S100 calcium-binding protein A12) in monocytes, whereas *IRF8* (interferon regulatory factor 8) and *CD83* (nuclear receptor subfamily 4, group A, member 3) in DCs. However, some known marker genes were not detected, such as FCER1A, which is considered a gene marker for cDC2. *FCER1G*, a related gene coding for the gamma chain of the high-affinity receptor for the Fc fragment of IgE (FCER), was detected as a DEG in all cell types except for CD8 T cells (Table S2).

We further confirmed the above cell type assignments using two other methods: Azimuth (Fig. 3A) and SingleR (Figure S3B). With Azimuth [24], we generated cell-type annotation results at three resolutions: low, medium, and high (Figure S3A, Table S3). As shown in Figure S3A, we detected Treg, TEM, and TCM cells, as well as naïve and memory B cells. Additionally, we assigned cell types using SingleR [25] and the human cell reference datasets, Blueprint and Encode (Table S4). By combining all three cell assignment efforts, consistent assignment results were demonstrated across three different methods (Tables S2, S3, and S4), where the main cell types were CD4 T cells and CD8 T cells for C0, B cells, and DCs for C1, monocytes for C2 and C5, and NK cells and CD8 T cells for C4 (Fig. 1B). We also successfully assigned CD14 monocytes and CD16 monocytes in C2 using SignleR (Figures S2A) or Azimuth (Figures S3A), separately.

### Cross-species comparison

To verify our cell clustering and assignments, we compared results between the cattle and human PBMCs. We downloaded the scRNA-seq dataset of the human PBMC from the GSE96583 [14, 15] and performed a joint Seurat clustering analysis with our Control cattle PBMC sample [22]. Plotting the single-cell transcriptomes via UMAP projection yielded largely overlapping distributions of cells from cattle and human samples (Figures S4A, B, and C), validating our scRNA-seq data generation, processing, clustering, and cell type assignment. With Azimuth [24], we obtained 13,601 individual cell transcriptomes of eight-cell types from the two samples (Figure S5A and B). The UMAP plot distribution reflected that the main cell types were CD4 T cells, CD8 T cells, B cells, NK cells, monocytes, DCs, and other minor populations (Figure S5A), confirming the seven cell types identified in our cattle Control sample. We also calculated the correlation between paired clusters of humans and cattle based on the top 2000 variable gene expressions. We showed the correlations were higher than 0.4 between humans and cattle, indicating a high similarity of these two species (Figure S4D). In summary, the analysis produced seven major cell types and their corresponding subtypes: CD4 T cells (CD4 Naïve, CD4 TCM, CD4 TEM, and Treg), CD8 T cells (CD8 Naïve, CD8 TCM, and CD8 TEM), B cells (B intermediate, B memory, B naïve, and plasmablast), monocytes (CD14 Mono and CD16 Mono), NK cells, ILCs, and DCs (cDC and pDC) (Fig. 1B). We will focus on these seven cell types for the subsequent sections unless specified otherwise.

### Cell cycle analysis for PBMC

**Fig. 2:**
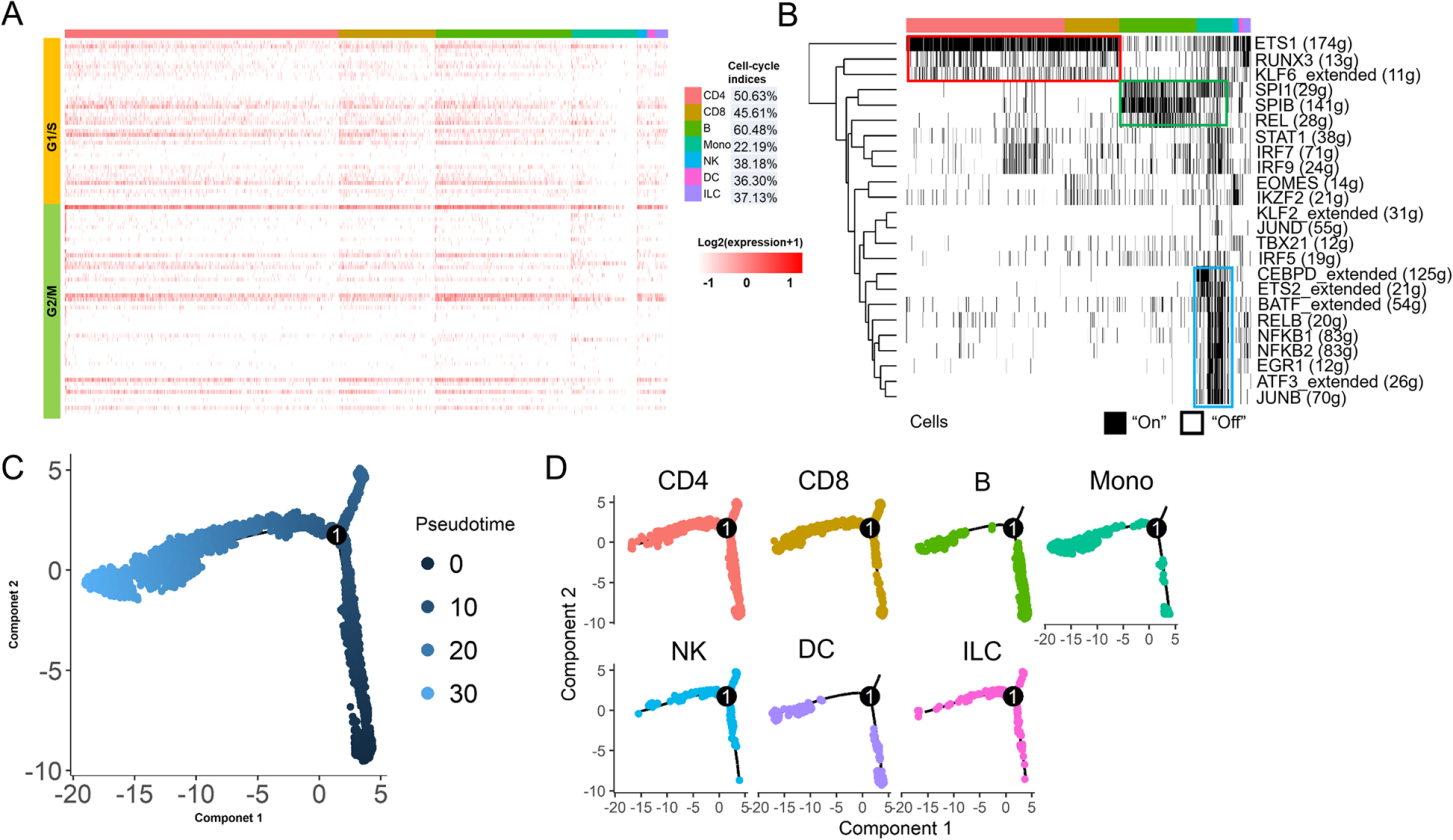
Cell-cycle, SCENIC, and Pseudotime analyses. **A** Cell-cycle analysis. Heatmap showing expression levels of cell-cycle-related genes in each cell type. Cells were ordered according to the average expression level of cell-cycle-related genes. The color key from white to red indicated expression levels from low to high. The cell-cycle index of each cell type is shown at the right. **B** SCENIC results. SCENIC binary regulon activity matrix showing all correlated regulons that were active in at least 1% of all regulons. Each column represents a single cell, and each row represents one regulon. The “regulon” refers to the regulatory network of TFs and their target genes. “On” indicates active regulons; “Off” indicates inactive regulons. Cluster labels correspond to those used in the UMAP plot. Representative transcription factors are highlighted. All cells (**C**) or individual cell type (**D**) pseudotime analysis using Monocle 2 for cell transcriptomes. Solid black lines indicate the main diameter path of the minimum spanning tree (MST) and provide the backbone of Monocle’s pseudotime ordering of the cells

We performed the cell cycle analyses to calculate their cell cycle indices (i.e., the ratio of actively proliferating cells of each feature, such as different samples and different developmental stages) and explore cell proliferation status, using sets of 43 G1/S and 55 G2/M genes (Table S5). The expression profiles of cell cycle-related genes revealed that the cell cycle indices were 50.63%, 45.61%, 60.48%, 22.19%, 38.18%, 37.13%, and 36.30% for CD4 T cells, CD8 T cells, B cells, monocytes, NK cells, ILCs, and DCs, respectively (Fig. 2A). Over LPS treatment time points, we found that monocyte cell cycle indices were 3.61%, 2.30%, 1.91%, and 31.88% in Control, 2 h-LPS, 4 h-LPS, and 8 h-LPS, respectively (Figure S6A). The cell cycle indices revealed that monocyte cell cycle progression was upregulated, suggesting that monocyte proliferation was dramatically activated during the early LPS treatment.

### Transcription factor analysis for PBMCs

To understand LPS-induced transcriptional activities of PBMC transcription factors (TF), we performed a transcription factor analysis using SCENIC [26] to identify regulators and gene regulatory networks. Through this analysis, we identified 24 active regulons in cattle PBMCs (Fig. 2B). Most of the regulons are related to immune functions in the differentiation and proliferation of T cells and B cells (ETS1, RUNX3, KLF6, SPI1, SPIB) or involved in mediating immune and inflammatory responses (REL, STAT1, IRF7, IRF9, EOMES, IKZF2, KLF2). The count range of target genes of these regulons was between 11 and 174 (Table S6). SCENIC analysis revealed several critical transcriptional regulators modulating cell type-specific gene regulatory networks. For all PBMCs, especially in CD4 T cells, CD8 T cells, B cells (to a lesser extent), monocytes, NK cells, DCs, and ILCs, we identified several universal TFs like ETS1, RUNX3, and KLF6_extended, as shown in Fig. 2B (red rectangle). We detected PU.1/SPI1, SPIB, and REL, primarily in B cells and monocytes (Fig. 2B, green rectangle). In CD4 T cells, CD8 T cells, B cells, monocytes, NK cells, and DCs, specific TFs, such as IRF5, IRF7, IRF9, and STAT1, were identified. For monocytes, we further identified their specific TFs, including CEBPD_extended, ETS2_exteneded, BATF_extended, IKZF2, NFKB1, NFKB2, and RELB, EGR1, ATF3_extended, and JUNB (Fig. 2B, blue rectangle). Therefore, TFs, as essential regulators of gene expression, are also marker genes for identifying cell types.

### Pseudotime analysis

To understand the developmental states of monocytes and DCs, we conducted a pseudotime analysis to infer cell trajectories using Monocle 2 [27]. Following a “developmental/transitional” path according to their transcriptomic similarity, we identified one significant and long-trajectory branch, with which cells are ordered in an arrangement from proximal to distal distribution (Fig. 2C). Combining with the pseudotime values (Table S7), we observed that the long-trajectory tree rooted from the bottom right to the top left, covering CD4 T cells, CD8 T cells, B cells, NK cells, ILCs, monocytes, and DCs (Fig. 2D). Larger portions of monocytes and DCs were observed at the top left end of the trajectory. The path also appeared to agree with our Seurat cluster results, i.e., monocytes in C2 and C5, while DCs in C6. Thus, those monocytes and DCs with the highest pseudotime scores might represent their terminal developmental states.

### Co-expression analyses

**Fig. 3:**
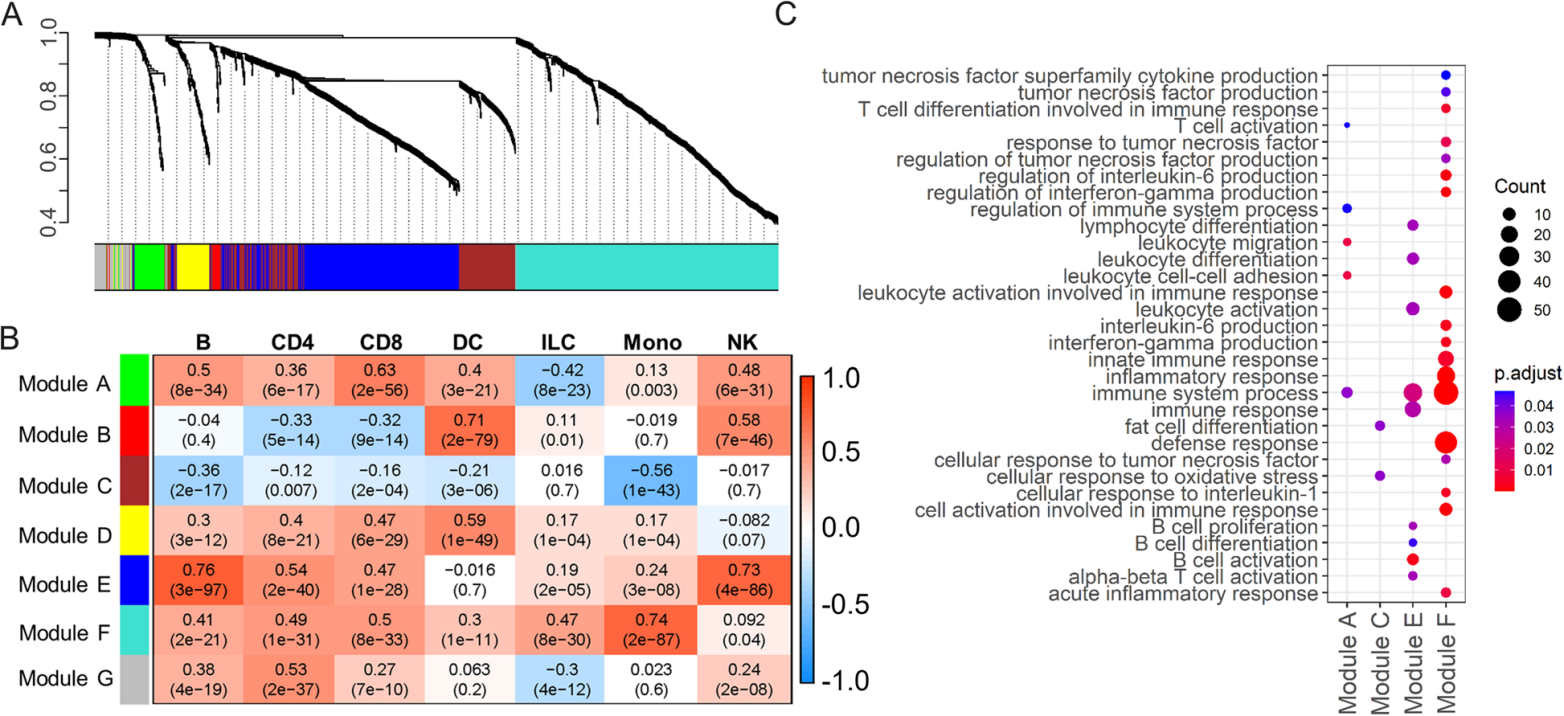
Co-expression analyses. **A** Dendrogram showing the gene co-expression network constructed using WGCNA. The color bar labeled as “Module colors” beneath the dendrogram represents the module assignment of each gene. **B** The relationship between modules and cell type. The upper numbers within each grid are the correlation between each module and cell type. The numbers in brackets represent the *p* values. **C** Selected significantly enriched GO terms based on genes within each module

To systematically investigate the genetic program dynamics, we performed a weighted gene co-expression network analysis (WGCNA) [28] using the top 2,000 marker genes reported by Seurat. Seven gene modules were identified by WGCNA (Fig. 3A), each containing gene sets that tend to be co-expressed (Table S8). To assign co-expressed gene functions to cell types, we calculated the correlation between each module (module eigengene) and each cell type (UMI) and generated a correlation heatmap in Fig. 3B. We then performed GO analyses for genes in each module to investigate their biological functions (Fig. 3C, Table S9). For example, Module E genes (blue) were enriched for immune responses, lymphocyte activation, differentiation, proliferation, and migration, especially with B cells and alpha–beta TCR T cells. Module E was also more correlated with B cells, NK cells, CD4 cells, and CD8 T cells. Module A genes (green) were enriched for the G protein-coupled receptor signaling pathway, kinase regulator activity, chemokine-mediated signaling pathway, regulation of chemotaxis, leukocyte adhesion and migration, regulation of cell death, calcium ion transport, and T cell activation. Module A was more correlated with CD8 T cells, B cells, NK cells, and CD4 cells. Module F genes (turquoise) were enriched for multiple GO terms, including (1) cellular response to LPS, LPS-mediated signaling pathways, innate immune responses, regulation of adaptive immune responses, leukocyte differentiation and adhesion, regulation of CD4 + alpha–beta TCR T cell activation, T-helper cell differentiation, macrophage migration, positive regulation of cytokine production, regulation of cell death; (2) positive regulation of interferon-γ production, positive regulation of interleukin-6 production, regulation of interleukin-1ß production and response, cellular response to fibroblast growth factor (FGF) stimulus, p38 mitogen-activated protein kinases (MAPK) cascade, and tumor necrosis factor (TNF) superfamily cytokine production; and (3) cell chemotaxis, response to reactive oxygen species, positive regulation of endopeptidase activity, response to glucocorticoid, collagen metabolic process, regulation of translation and transcription, lysosome, and osteoclast differentiation. Module F was mainly correlated with monocytes, followed by CD8 T cells, CD4 T cells, ILCs, and B cells (Fig. 3C, Table S9). Therefore, our co-expression analyses identified critical gene sets corresponding to cell type-differential functions.

### Marker gene expression for PBMC clusters

**Fig. 4:**
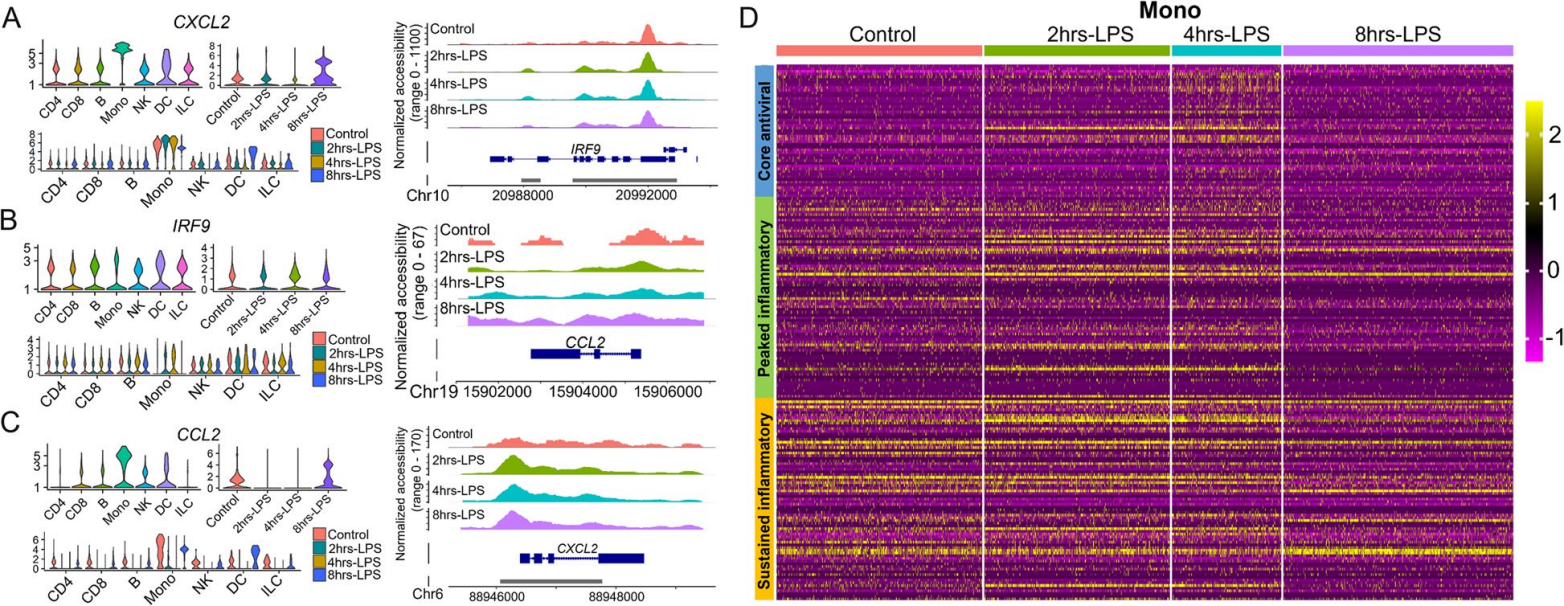
Specific gene expression responses of innate immunity induced by lipopolysaccharide in cattle PBMC. Gene expressions of *CXCL2* (**A**), *IRF9* (**B**), and *CCL2* (**C**) in seven cell types, four PBMC samples of different treatment time points, or across their combinations. On their right, the changes of chromatin accessibility peak profiles near these three gene promoters over the treatment time course were derived from scATAC-seq. **D** Heatmap showing scaled expression levels of three gene modules (core antiviral, peaked inflammatory, and sustained inflammatory) in monocytes

Marker gene expression analysis was aimed to determine the expressions of essential known marker genes and their nearby chromatin accessibilities in several cell types. Based on the Seurat results, we obtained distinct sets of marker genes among these cell types (Table S2). For example, *CXCL2* (C-X-C motif ligand 2) expression was higher in monocytes than others (Fig. 4A). When we analyzed cell type-specific responses over time, we found that *CXCL2* expression was higher in monocytes than other cell types; its expression was elevated in Control, 2 h-LPS, and 4 h-LPS samples decreased in 8 h-LPS. Correspondingly, we also detected increased levels of chromatin accessibility in the *CXCL2* promoter (Fig. 4A). A similar pattern was also found for *CXCL5* (Figure S7B). When we plotted individual or combined marker gene expression over time, *IRF9* was expressed higher in DCs than other cell types (ANOVA test, *p* < 2 × 10^–16^). However, due to the small cell count of DCs, we did not detect significant differences in gene expression or chromatin openness over time points (Fig. 4B). *CCL2* (C–C motif ligand 2, encoded by the negative-sense strand) expression was higher in Control and 8 h-LPS than 2 h-LPS and 4 h-LPS, which were in line with higher chromatin accessibility in Control and 8 h-LPS (Fig. 4C). Also, in monocytes, we detected *IL1B* expression, which was decreased from early (2 h-LPS, 4 h-LPS) to late time points (8 h-LPS), while in DCs and ILCs, *IL1B* expression was increased (Figure S7A). Hence, we found a consistent correlation between expression and chromatin accessibility for selected marker genes.

### Gene expression patterns during LPS treatment

In humans, Shalek et al. [13] used the single-cell gene expression profiles to partition the LPS-responsive genes into two programs: the antiviral programs and the inflammatory programs, which include three modules: the core antiviral module (enriched for annotated antiviral and interferon response genes), the peaked inflammatory module and the sustained inflammatory module. We obtained these three human LPS-responsive gene lists and plotted the expression patterns of the bovine ortholog genes from monocytes with or without LPS treatment (Fig. 4D). The analysis showed that the gene expression sustained until four hours post LPS treatment for the sustained inflammatory module and then decreased slightly at eight h. But for the core antiviral module and the peaked inflammatory module, gene expression was increased from Control to 4 h-LPS and fell to Control levels in 8 h-LPS. These results were consistent with the observation in human PBMCs that the antiviral and inflammation responses mainly occurred early but decreased in the late-stage [13]. Therefore, we observed similar gene expression patterns for those three modules in cattle and humans during LPS treatment.

### Trait-relevant cell clusters

**Fig. 5:**
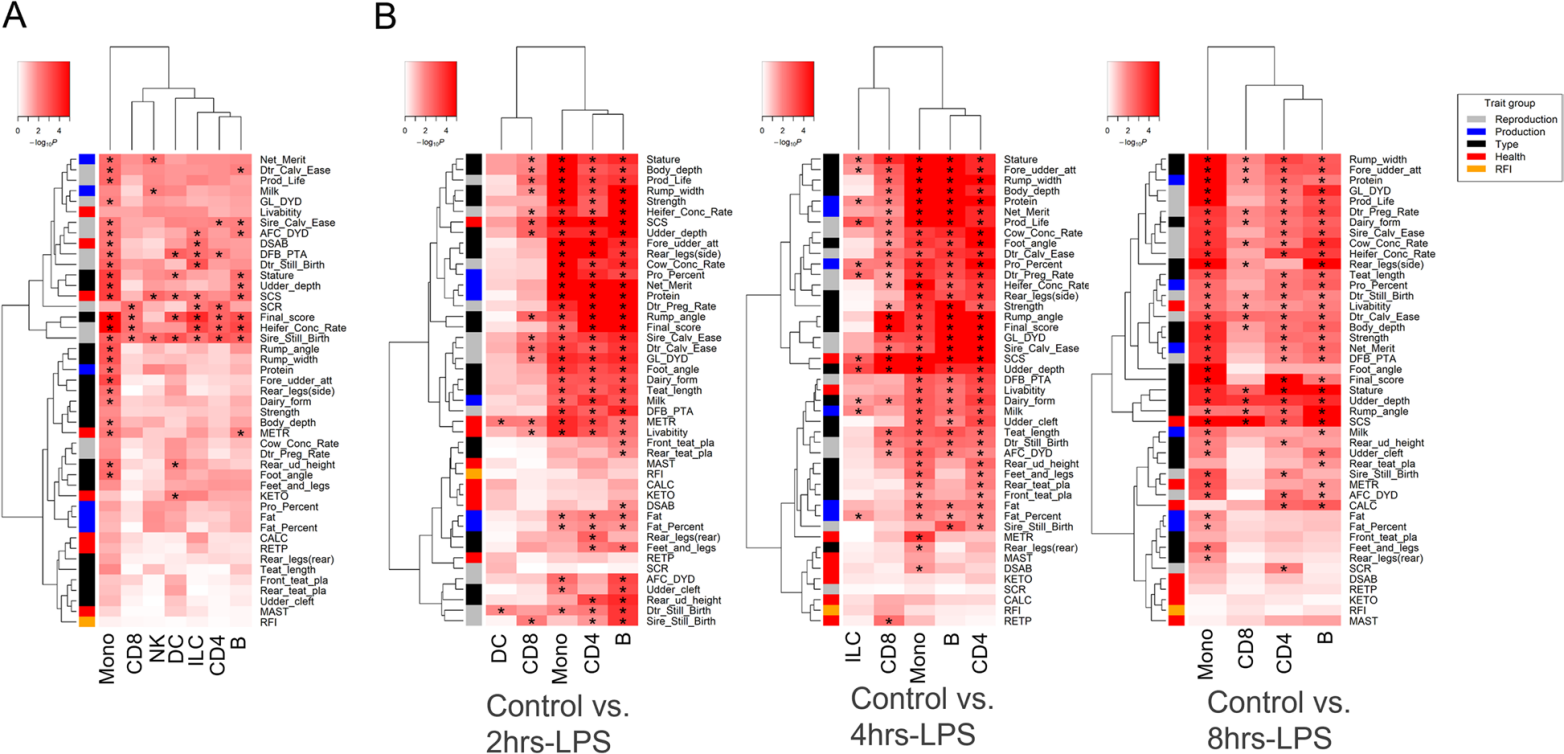
Associations of cell clusters with complex traits based on GWAS signal enrichment analyses using DEGs/marker genes among cell types (**A**) and among cattle PBMC LPS-treatment samples (top 5%) (**B**). “*” denotes FDR < 0.05

Using edgeR [29], we detected thousands of marker genes among seven cell types (Table S10-S16). Using a permutation-based marker-set test approach (Methods), we tested the enrichment of 45 GWAS signals within these marker genes of distinct cell types (FDR < 0.05) (Fig. 5A). Reproduction traits were significantly associated with all cell types, reflecting the potential functions of these cell types related to fertility and tissue development. Since all cell types in the present study were immune cells, their high correlation with reproduction traits confirmed our previous findings [30]. Additionally, health traits, such as SCS (somatic cell score, an indicator of mastitis), were associated with most cell types, confirming that these cell types have a role in immunity and tissue integrity. Body conformation traits were also significantly associated with monocytes.

Moreover, based on the marker genes reported by edgeR between cell clusters across the LPS-untreated (Control) and LPS-treated (2 h-LPS, 4 h-LPS, and 8 h-LPS) PBMC samples, we also detected similar results (Fig. 5B). In all three comparisons, we found that the cell types with the most DEGs were monocytes, CD4 T cells, and B cells. Generally, all cell types were significantly associated with reproduction, body conformation, and health traits. In both Control vs. 2 h-LPS and Control vs. 4 h-LPS comparisons, monocytes were associated with heath traits, especially immune traits, such as SCS and Livability, but not with the health traits relating to metabolic diseases.

## Discussion

In the current cattle single-cell analyses, we successfully detected and confirmed seven major cell types (including CD4 T cells, CD8 T cells, B cells, monocytes, NK cells, ILCs, and DCs), as well as their responses to LPS challenge in vitro using scRNA-seq and scATAC-seq. We characterized these cells and their genes in detail. Our bioinformatic analyses indicated that LPS could increase PBMC cell cycle progression, cellular differentiation, and chromatin accessibility. Our gene analyses further showed significant changes in differential expression, transcription factor binding site, gene ontology, and regulatory interactions during the PBMC responses to LPS. These results of cattle PBMC generally agreed with the existing human and cattle studies [2, 13, 16]. The reactions to LPS treatment include innate immunity activation of monocytes and dendritic cells, featuring the antiviral program mediated by interferon regulatory factors (IRFs) and the inflammatory program mediated by NF-κB and pro-inflammatory cytokines such as CCL2 and CXCL2. LPS induced activation of monocytes and dendritic cells, likely through their upregulated TLR4 receptor. NF-κB was observed to be activated by LPS and increased transcriptions of an array of pro-inflammatory cytokines, in agreement that NF-κB is an LPS-responsive regulator of innate immune responses.

For example, our transcription factor analysis discovered crucial TFs, like NFKB1, NFKB2, RELB, and others for monocytes (Fig. 2B blue rectangle). We also compared the expression patterns of NFKB1 and NFKBIZ (Figure S7C and D). NFKB1 displayed a universal gene expression pattern in all cell types over all time points, while NFKBIZ was mainly detected in monocytes, DCs, and ILCs, especially in 2 h-LPS and 4 h-LPS. It is generally accepted that NF- κB is a known pleiotropic TF present in almost all cell types and is involved in many biological processes such as inflammation, immunity, differentiation, cell growth, tumorigenesis, and apoptosis. Moreover, we found other TFs, such as IRF5, IRF7, IRF9, and STAT1 (Fig. 2B). Earlier bulk studies have shown that IRF5, IRF7, and IRF9 belong to the interferon response factor (IRF) family. After activation via the JAK-STAT signaling pathway, these TFs bind specifically to the interferon consensus sequence (ICS) in the upstream promoters [31] and regulate transcription of interferons and inflammatory cytokines [32]. They control many aspects of innate and adaptive immune responses, including responding to pathogens to induce pro-inflammatory responses and regulating immune cell differentiation. Therefore, our single-cell analyses confirmed the previous bulk study results of these critical TFs. In our DEG analyses, we pinpointed many factors like monocyte chemotactic protein-1 (CCL2) and monocyte chemotactic protein-3 (CCL7), which can regulate the chemotaxis and other functions of monocytes [33]. CCL2 is a chemokine that belongs to the CC chemokine family [34]. CCL2 is also called monocyte chemoattractant protein 1 (MCP1) and small inducible cytokine A2. CCL2 recruits monocytes, memory T cells, and dendritic cells to the sites of the inflammation [35]. CXCL2 is another small cytokine belonging to the CXC chemokine family. It activates cells via binding to a cell surface chemokine receptor CXCR2 [36].

Additionally, our previous studies using bulk RNA-seq data demonstrated that the immune system was significantly associated with many health and fertility traits in the cattle [30, 37]. This study further detected trait-relevant cell types by integrating LPS-induced DEGs with large-scale GWAS of 45 complex traits in Holstein. We found that selected DEGs were significantly associated with immune-relevant health, milk production, and body conformation traits.

### Limitations and future directions

Some essential marker genes are not detected in this study. These can be due to methodological noise, where a gene is expressed but not detected by the sequencing technology, and/or due to the biological absence of expression. Moreover, we did see discrepancies in cell-type assignments using different methods. For example, SingleR assigned C5 and C6 as monocytes, while marker gene expressions and Azimuth annotated them as DCs and macrophages. These are not surprising partially because monocytes, some DCs, and macrophages are closely related, such that in silico predictions may not be reliable. We also checked the relative portion changes among the seven cell types across different time points during LPS treatment (Fig. 1C). We found monocytes decreased first from 11.42% in Control to 7.96% in 2 h-LPS and 6.33% in 4 h-LPS and then increased to 29.21% in 8 h-LPS (Table S1). This corresponded to that monocyte’s cell cycle index increased over the LPS treatment time course. However, it is noted that these cell number changes were from one-time measurement and may be impacted by the Azimuth cell type assignment. T cells also changed gene expression and cell activation, resulting from bystander effects secondary to the monocyte response. In addition, T cells may respond to LPS because a recent report shows that TLR2/4 are expressed by bovine T cells [26]. There are also known differences in PBMCs of these two mammalian species, which we did not detect. For example, besides common α and β T cells, γ and δ T cells typically represent 1–10% of circulating T lymphocytes in adult human individuals and approximately 10–25% in adult cattle. This number can be as high as 40% in the young calves [38]. Previous work has also shown that human and bovine γδ T cells can be directly activated by LPS, suggesting an innate role of γδ T cells [39]. We were unable to demonstrate a sufficient number of γδ T cells for analysis in this study because adult cattle have much lower levels of circulating γδ T cells. Our ability of γδ T cell assignment was also undermined, probably because we used human reference cell types to assign cattle cells. These designations might be biased towards human-specific features and functions. Therefore, more dedicated experiments are warranted to investigate the roles of ruminant-specific γδ T cells in cattle.

## Conclusions

The functional results inferred from these single-cell-based data sets were consistent with previous findings. They revealed new findings in LPS-driven cell proliferation and differentiation, differential gene transcription, and correlation between DEGs and production traits in cattle. Single-cell analyses provide an unprecedented opportunity to dissect cell lineages and heterogeneity and understand their identity, differentiation, and function. The successful applications of these new technologies in farm animals like cattle indicated that some research bottleneck problems could be alleviated, e.g., only limited immunological reagents are available in cattle. This study provides an initial example for cattle single-cell analysis. It opens the door for discoveries about the roles of cell types and marker genes in complex traits at single-cell resolution.

## Materials and methods

### Sample collection

All samples were collected with the approval of the Dairy Cattle Research Centre in Shandong Academy of Agricultural Sciences under Protocol 20–123, and all experiments were carried out in compliance with the ARRIVE guidelines.

Four 2-year old Holstein female lactating cattle were used for blood collection from the tail vein in Jinan Jiabao Dairy Co., Ltd. After pooling; four whole blood samples included either no LPS treatment—control sample CO, or three treated samples with LPS (2 μg/ml, Product Number: L2880, Sigma-Aldrich, Saint Louis, MO, USA) for 2 h (2 h-LPS), 4 h (4 h-LPS), and 8 h (8 h-LPS) at 37 °C. PBMCs were isolated by centrifugation of whole blood on Hanks’ Balanced Salt Solution (Solarbio; Beijing, China) at 500 g for 20 min at room temperature.

### Single-cell isolation, scRNA-seq, and scATAC-seq library preparation and sequencing

After cell isolation, scRNA-seq Library for 10 × Genomics v3 chemistry was generated following the Chromium Single Cell 3’ Reagent Kits v3 User Guide: CG000183 Rev C. In brief, cells were barcoded and mixed with reverse transcriptase into a Gel Beads-In-Emulsions (GEMs), then R1 (read 1 primer sequence) was put into the molecules during GEM incubation. P5, P7, a sample index, and Read 2 primer sequence were included during library construction via end repair, A tailing, adaptor ligation, and PCR. The final libraries containing the P5 and P7 primers were used in Illumina bridge amplification.

For scATAC-seq, PBMC nuclei were prepared for library preparation sequencing. Library generation was accomplished following the Chromium Single Cell ATAC Reagent Kits v1.1 User Guide: CG000209 Rev D. Concisely, Nuclei suspensions were incubated in a Transposition Mix that includes a Transposase, which preferentially fragmented the DNA in open regions of the chromatin. Instantaneously, adapter sequences were added to the ends of the DNA fragments. Nuclei were barcoded into a Gel Beads-In-Emulsions (GEMs), a sample index, P7, and Read 2 sequence were added during library construction via PCR. In the same way, the scATAC-seq libraries contained the P5 and P7 primers used in Illumina bridge amplification. Finally, scRNA-seq and scATAC-seq libraries were sequenced on the Illumina Novaseq 6000 platform (Illumina, San Diego, CA, USA) with double-end 150 bp.

### Generation of single-cell transcriptomes

10X Genomics raw data were handled by the Cell Ranger Single-Cell Software Suite (release 3.1.0) and Cell Ranger “*mkfastq*” was used to demultiplex raw base-call files into FASTQ files followed by using Cell Ranger “*count*” to perform alignment, filtering, barcode counting, and UMI counting. Using default parameters, the raw reads were aligned to the ARS-UCD1.2 cattle reference genome [40] by Cell Ranger “pipeline.” using default parameters. The results are summarized in Supplemental Table S1. All downstream single-cell analyses were accomplished using the Seurat 3.2 [22] R package v3.6.3.

### Quality control, dimension reduction, and cell clustering

Seven thousand one hundred seven (Control), 9,174 (2 h-LPS), 6,741 (4 h-LPS), and 3,119 (8 h-LPS) cells passed the quality control thresholds. All genes expressed in fewer than three cells were removed. The cut-off of the number of gene expressions per cell was set at 200 as low and < 3,000 as high; UMI counts less than 200; the percent of mitochondrial-DNA derived gene-expression < 20%. LogNormalize method of the "Normalization" function was used to determine the expression value of genes. We then constricted the corrected expression matrix to the subsets of HVG, centered, and scaled values before performing dimension-reduction and clustering. We selected 2,000 genes as HVG using the “*FindVariableFeatures*” function with default parameters. The “*RunPCA*” function was used to perform the principal components analysis (PCA) on the single-cell expression matrix with genes restricted to HVG. Using a permutation test implemented by the “*JackStraw*” function, we determined the number of significant principal components (PC). The top 12 PCs were used for clustering and UMAP analysis. The weighted Shared Nearest Neighbor (SNN) graph-based clustering method executed by the “FindNeighbors” function was used to find clusters. We utilized the “*FindClusters*” function to conduct the cell-clustering analysis by inserting cells into a graph structure in the PCA cluster. Based on the number of cells in our study, we set the parameter resolution to 0.05. Visualization of the cells was performed using the UMAP algorithm as implemented by the Seurat “*RunUMAP*” function. With default parameters, canonical cell-type marker genes maintained across conditions were identified using the “FindConservedMarkers” function.

### Assigning cell type labels to single-cell clusters

We utilized two methods to label the cell clusters identified by Seurat. First, we projected the PBMC data onto an annotated PBMC CITE-Seq reference dataset [41] using Azimuth [24]. Each cell received an assignment and prediction score to a cell class in the reference. We normalized data using the “*SCTransform*” function [42] and then found anchors between reference and query using “*FindTransferAnchors*.” Here we used a precomputed supervised PCA (spca) transformation. We then transferred cell type labels and protein data from the reference to the query using “*MapQuery*.” Additionally, we used SingleR [25] to annotate raw expression data for the filtered cells with default parameters using the Blueprint [43] and Encode [44] human cell atlases.

### Pseudotime trajectory analysis

For trajectory analysis, we used Monocle 2 [27] to order cells in pseudotime based on their transcriptional similarities, with UMI counts modeled using a negative binomial distribution. First, we integrated the preprocessed Seurat objects into Monocle 2 utilizing the “*newCellDataSet*” function. We then determined the differentially expressed genes or marker genes using the “*differentialGeneTest*” function. We next reduced the dimensionality of the data to two dimensions using the discriminative dimensionality reduction with trees (DDRTree) method implemented in the “*reduceDimension*” function. Finally, after pseudotime calculations were made for each cell, we projected clusters derived from the Seurat object onto the minimum spanning tree upon cell order using the “*plot_cell_trajectory*” function.

### Cell-cycle analysis

Sets of 43 G1/S and 55 G2/M genes [45] were used in the cell-cycle analysis. To calculate the ratio of actively proliferating cells of each feature, such as different clusters and different time points, we first calculated the total expression levels of all 98 cell-cycle genes in every single cell, and only cells with mean expression levels higher than the average values of all clusters were regarded as actively proliferating.

### Single-cell regulatory network inference and clustering (SCENIC) analysis

We conducted SCENIC analysis on cells after filtering for each major cell type using the R package SCENIC v1.1.2 [26], a computational workflow that predicts TF activities from scRNA-seq data. Briefly, SCENIC infers co-expression modules between TF and candidate target genes using machine learning regression techniques (e.g., random forest or gradient boosting machines), pruned based on the enrichment of the TF motif around the TSS of the potential target genes, resulting in regulons. Based on the AUCell algorithm, SCENIC calculates each regulator’s activity in single-cell transcriptomes to obtain the corresponding area under the curve (AUC) scores, which are used to rank the cells for a given regulon and determine a threshold for active or inactive expression. Then the network activity was converted into ON/OFF, thus making the final output binary (binary regulon activity matrix). Individual regulons were constructed from the scRNA-seq data. Regions for TF searching were restricted to a 10 kb distance centered on the transcriptional start site (TSS) or 500 bp upstream of the TSS. First, TF-gene co-expression modules were defined in a data-driven manner with GENIE3 v1.8.0. Subsequently, those modules were refined via RcisTarget by keeping only those genes that contain the respective transcription factor binding site (TFBS). Once the regulons were constructed, the method AUCell scored individual cells by assessing for each TF separately whether target genes were enriched in the top quantile of the cell signature.

### Weighted gene co-expression network analysis

WGCNA was performed with functions in the WGCNA v1.69 R package following the previously published study by Tosches and colleagues [46]. According to the methods, the analyses were performed on pseudocells, calculated as averages of 100 cells randomly chosen within each cluster. DC was not included due to its small cell number. The top 2,000 highly variably expressed genes determined in Seurat were used for analysis. Briefly, the topological overlap matrix (TOM) was constructed with softPower and was set to 2. The hub genes for each module were identified as module eigengene. The GO enrichment analysis was performed by ClusterProfiler [47] R package using hub gene data sets, and the Benjamini–Hochberg method was employed for multiple test correction. GO terms with a *P*-value lower than 0.05 were considered as significantly enriched.

### Gene differential expression analysis

To get the lists of marker genes, we first extracted the genes’ UMIs across cells within each cluster and then assigned cells to each sample. Based on the gene × cells matrix, we utilized edgeR [29] to detect DEGs for each cluster in each pairwise comparison among Control, 2 h-LPS, 4 h-LPS, and 8 h-LPS (Tables S10-15).

### Single-cell ATAC-seq alignment and data processing

For scATAC-seq analyses, we aligned the sequence using the 10 × Genomics Cell Ranger ATAC pipeline (version 1.2) against the UCD-ARS1.2 genome. The “*Cell Ranger Aggr*” function normalizes the number of confidently mapped reads per cell across the libraries. We processed the data with Seurat and the additional package Signac (v1.1.0) [48]. We first computed quality control (QC) metrics and removed the cells with the number of expressed genes < 500. We then normalized the filtered data by the “*RunTFIDF*” function and removed features in less than 20 cells with the “*FindTopFeatures*” function. We next ran singular value decomposition (SVD) using “*LSI*” with the features selected above. Next, we performed graph-based clustering by “*FindNeighbors*” and “*FindClusters*” functions using the first 30 dimensions of reduction as an input. Finally, the read coverage of regions near specific genes in each group was plotted by the “*CoveragePlot*” function. On average, 3,798 fragments per cell were obtained, and 4,200 cells were recovered.

### GWAS signal enrichment analysis

Details of the single-marker GWAS and fine-mapping analyses designed for the body type, reproduction, and production traits from 27,214 U.S. Holstein bulls, intended for health traits from 11,880–24,699 bulls, and feed efficiency (i.e., RFI) from 3,947 Holstein cows were previously reported [30, 49–51]. As the complex traits being explored were highly polygenic, the sum-based marker-set test methodology shown in Eq. 1 was utilized as in QGG package v1.0 [52] to establish whether GWAS signals were enhanced in marker genes of distinct cell clusters and DEGs of six pairwise comparison groups (Control vs. 2 h-LPS, Control vs. 4 h-LPS, Control vs. 8 h-LPS, 2 h-LPS vs. 4 h-LPS, 2 h-LPS vs. 8 h-LPS, 4 h-LPS vs. 8 h-LPS). We included 20-kb windows around gene regions to identify the potential *cis*-regulatory variants. Previous studies indicated that this method had at best equal power compared to other commonly used GWAS signal enrichment methods in humans [37, 53], *Drosophila melanogaster* [54], and livestock [55–57], especially for the highly polygenic traits.

1 $${\mathrm T}_{sum}={\textstyle\sum_{i=1}^{m_f}}b^2$$

In this equation, *m*_*f*_ is the number of genomic markers within a list of genes (marker genes of each cell cluster or DEGs from pairwise comparisons in each cell cluster), and *b* is the marker weight from single-marker GWAS. We restricted marker-set sizes and linkage disequilibrium patterns among markers by utilizing the genotype cyclical permutation strategy [52]. We first organized marker effects (i.e., ${b}^{2}$) utilizing their chromosome positions (i.e., ${b}_{1}^{2}$, ${b}_{2}^{2}$, ⋯ ${b}_{m-1}^{2}$, ${b}_{m}^{2}$). We then at random designated one marker (i.e., ${b}_{k}^{2}$) from this vector as the first place, and altered the remaining ones to new positions while retaining their original orders (i.e., ${b}_{k}^{2}$, ${b}_{k+1}^{2}$, ⋯ ${b}_{m-1}^{2}$, ${b}_{m}^{2}$, ${b}_{1}^{2}$⋯ ${b}_{k-1}^{2}$) to conserve LD patterns among markers. We determined a new summary statistic for an allocated list of genes using their original chromosome locations. To attain an empirical *P*-value for the list of genes, we went over this permutation procedure 10,000 times. We used a one-tailed test of the proportion of random summary statistics greater than that observed.

### Cross-species comparison

We downloaded a single-cell RNA-seq dataset of human PBMC from GSE96583. We first merged expression matrices of the two species (cattle and human) based on the intersection of the detected homologous genes. Next, we performed expression matrix preprocessing separately for the two species, followed by integrating three datasets using functions in Seurat v3.2 [22]. The top 13 PCs were selected, and the resolution was set to 0.18 to yield 13 cell clusters.

## Supplementary Information

**Additional file 1:** **Figure S1.** Cell clustering. **Figure S2.** Cell marker gene expression. **Figure S3.** Cell type annotation using Azimuth and SingleR (based on the human Blueprint and Encode cell atlas references). **Figure S4.** Comparative analyses between cattle and human PBMC. **Figure S5.** Comparative analyses between cattle and human PBMC under two resolutions using Azimuth. **Figure S6.** Cell cycle analysis for cattle PBMC. **Figure S7.** Gene expression of innate immunity genes and transcription factors during lipopolysaccharide treatments in cattle PBMC. **Figure S8.** Heatmap showing scaled expression levels of three gene modules (core antiviral, peaked inflammatory, and sustained inflammatory) in CD4 cells, CD8 cells, B cells, separately and jointly with monocytes.

**Additional file 2:** **Table S1.** Summary of scRNA-seq and scATAC-seq dataset. **Table S2.** Marker genes of each cell type. **Table S3.** Cattle PBMC cell type annotation under three resolutions using Azimuth. **Table S4.** Cattle PBMC cell type annotation using SingleR. **Table S5.** The expression of 93 cell cycle-related genes in each cell. **Table S6.** The summary information of TF identified by SCENIC. **Table S7.** Single cell’s pseudotime value obtained from Monocle2. **Table S8.** Gene list of each module identified by WGCNA. **Table S9.** Enrichment results of each module identified by WGCNA.

**Additional file 3:** **Table S10.** Differentially expressed genes with each cell cluster between CO and T1 identified by edgeR. **Table S11.** Differentially expressed genes with each cell cluster between CO and T2 identified by edgeR. **Table S12.** Differentially expressed genes with each cell cluster between CO and T3 identified by edgeR. **Table S13.** Differentially expressed genes with each cell cluster between T1 and T2 identified by edgeR. **Table S14.** Differentially expressed genes with each cell cluster between T1 and T3 identified by edgeR. **Table S15.** Differentially expressed genes with each cell cluster between T2 and T3 identified by edgeR. **Table S16.** Human and cattle PBMC cell type annotation under three resolutions using Azimuth.

## Data Availability

The accession number for the scRNA-seq data reported in this study is GEO: GSE166473. The GWAS summary statistics for all complex traits have been submitted to Figshare, i.e., body type, production, and reproduction traits under https://figshare.com/s/ea726fa95a5bac158ac1, and the remaining ones under https://figshare.com/s/94540148512dddf7ed32. All scripts and source codes can be found in the Supplemental Material and in https://github.com/YahGao/PBMC-scRNA-seq.

## Abbreviations

CCL2: C–C Motif Chemokine Ligand 2/Chemotactic Protein-1
CCL7: C–C Motif Chemokine Ligand 7/Chemotactic Protein-3
CD14: Cluster of differentiation 14
CD83: Nuclear receptor subfamily 4, group A, member 3
CXCL2: C-X-C motif ligand 2
DC: Dendritic cell
DEGs: Differentially expressed genes
FGF: Fibroblast growth factor
GO: Gene ontology
GRN: Gene regulatory network
HVG: Highly variable genes
ICS: Interferon consensus sequence
IκB: NF-κB inhibitor
IL-1: Interleukin-1
IRF: Interferon response factor
IRF8: Interferon regulatory factor 8
LPS: Lipopolysaccharide
MAPK: P38 mitogen-activated protein kinases
MCP1: Monocyte chemoattractant protein 1
MHC: Major histocompatibility complex
NF-κB: Nuclear factor kappa-light-chain-enhancer of activated B cells
NK: Natural killer
PBMC: Peripheral blood mononuclear cell
S100A12: S100 calcium-binding protein A12
scATAC-seq: Single-cell sequencing assay for transposase-accessible chromatin
scRNA-seq: Single-cell RNA sequencing
SCENIC: Single-Cell rEgulatory Network Inference and Clustering
SCS: Somatic cell score
STAT: Signal transducer and activator of transcription
TF: Transcription factor
TNF: Tumor necrosis factor
TNFα: Tumor necrosis factor-alpha
UMAP: Uniform Manifold Approximation and Projection
WGCNA: Weighted gene co-expression network analysis
