# Supplementary material for: Single-cell transcriptomic and chromatin accessibility analyses of dairy cattle peripheral blood mononuclear cells and their responses to lipopolysaccharide
Source: BMC Genomics. 2022 Apr 30;23:338. doi: 10.1186/s12864-022-08562-0 (PMC9063233; doi:10.1186/s12864-022-08562-0)
Supplement: Supplementary file 1 — Additional file 1: Figure S1. Cell clustering. Figure S2. Cell marker gene expression. Figure S3. Cell type annotation using Azimuth and SingleR (based on the human Blueprint and Encode cell atlas references). Figure S4. Comparative analyses between cattle and human PBMC. Figure S5. Comparative analyses between cattle and human PBMC under two resolutions using Azimuth. Figure S6. Cell cycle analysis for cattle PBMC. Figure S7. Gene expression of innate immunity genes and transcription factors during lipopolysaccharide treatments in cattle PBMC. Figure S8. Heatmap showing scaled expression levels of three gene modules (core antiviral, peaked inflammatory, and sustained inflammatory) in CD4 cells, CD8 cells, B cells, separately and jointly with monocytes. [file 12864_2022_8562_MOESM1_ESM.pdf]

Fig. S1

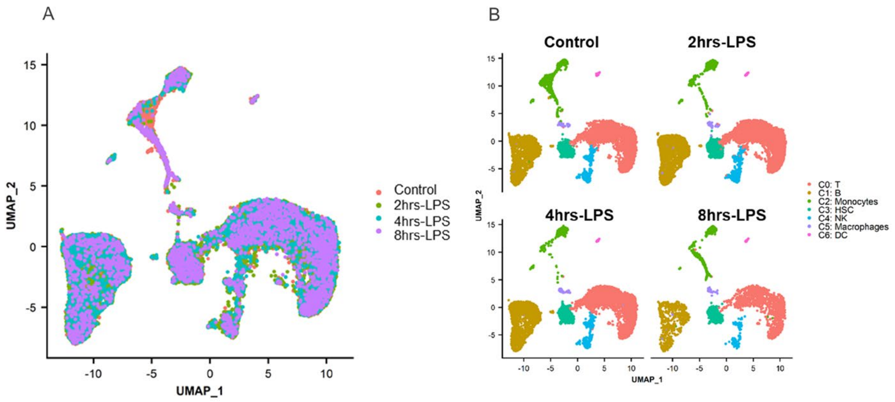

Figure S1. Cell clustering. (A) UMAP projection plot showing dimensional reduction of the distribution of 26,141 individual cell transcriptomes from four cattle PBMC samples, including no LPS treatment (Control), treated samples with LPS for 2 hours (2hrs-LPS), 4hours (4hrs-LPS), and 8 hours (8hrs-LPS). (B) UMAP projection plot showing annotation of four cattle PBMC samples, separately.

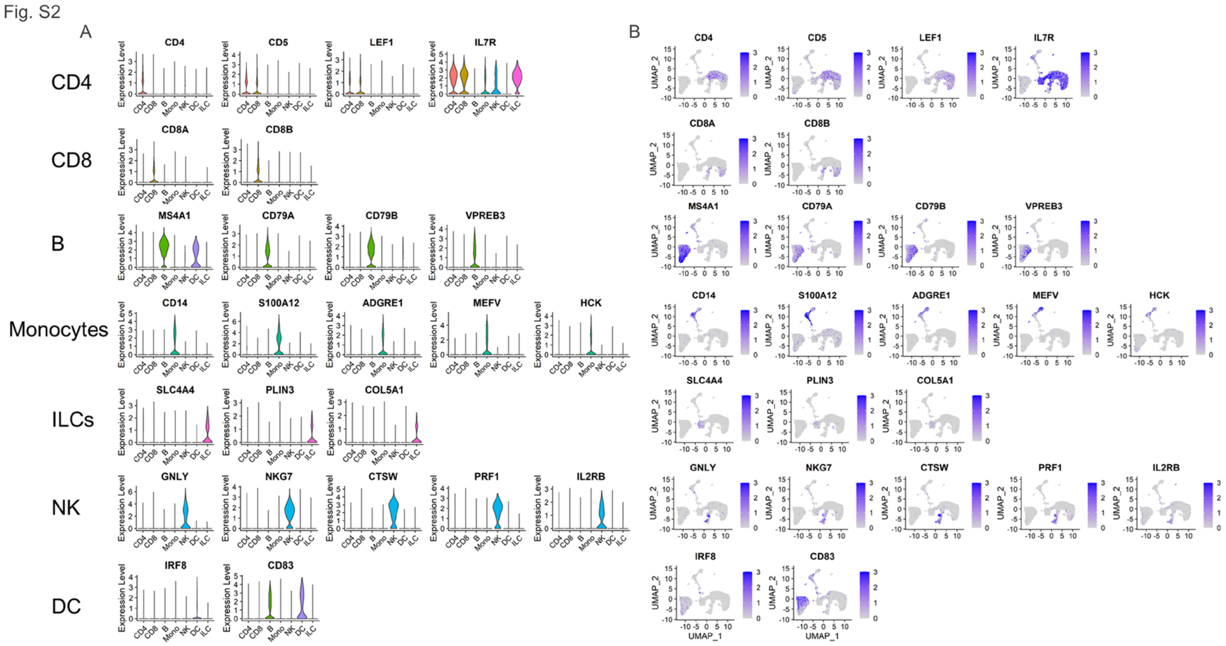

Figure S2. Cell marker gene expression. A. Violin plots of selected DEGs among cell clusters i.e. cell cluster-specific genes. Expression in each cell type is shown along with the probability density of gene expression, denoted by the shape of the plot. We chose these DEGs to assign a cell type to each cluster, also because they are known immune cell type marker genes. B. UMAP projection plots showing transcript accumulation for cell marker genes in individual cells. Color intensity indicates the relative transcript level for the indicated gene in each cell.

Fig. S3

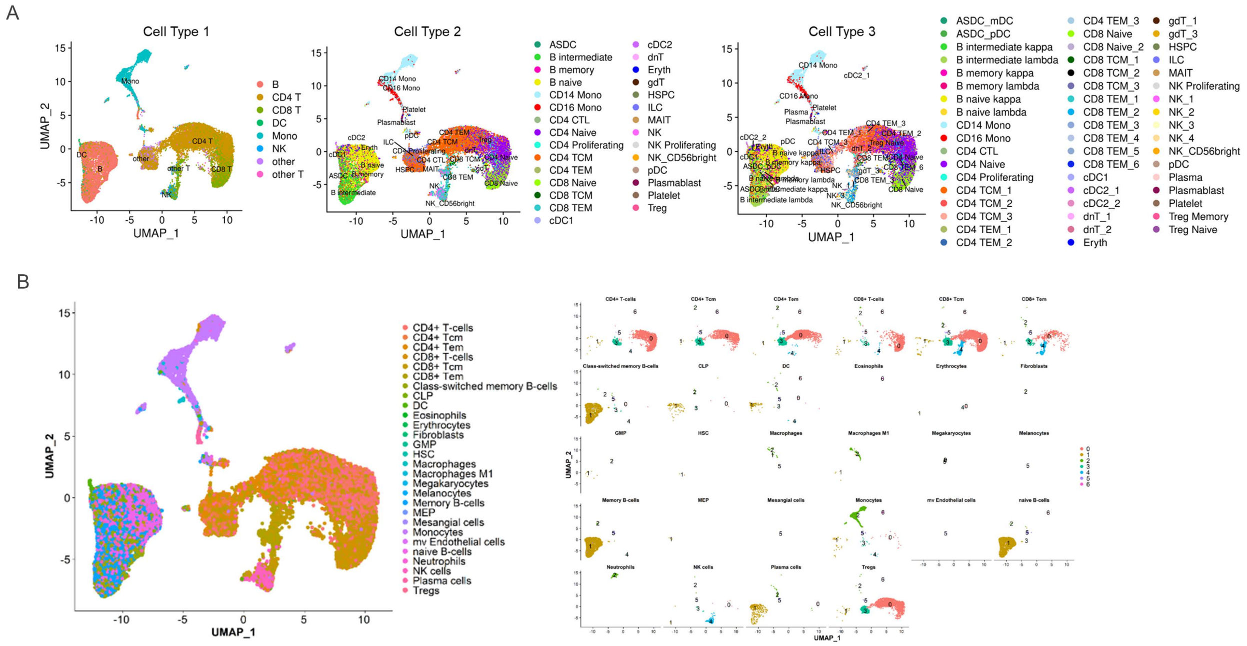

Figure S3. Cell type annotation using Azimuth and SingleR (based on the human Blueprint and Encode cell atlas references). A. UMAP projection plots showing the cell type annotation results under three resolutions using Azimuth. B. UMAP projection plot showing the annotation cell types of the 26,141 individual cell transcriptomes from the four cattle PBMC samples (left). UMAP projection plot shows the distributions of different cell types in seven cell clusters (right).

Fig. S4

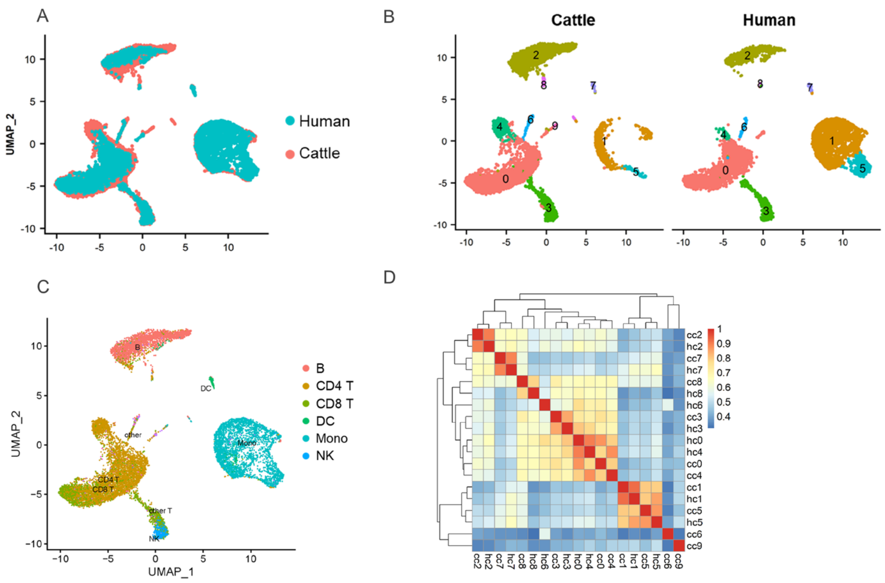

Figure S4. Comparative analyses between cattle and human PBMC. A. UMAP projection plot showing dimensional reduction of the distribution of 13,601 individual cell transcriptomes from our cattle control sample (red) and one human control PBMC sample (turquoise). B. UMAP projection plot showing 10 major clusters (labeled from 0 to 9) of the 13,601 individual cell transcriptomes derived from either cattle or human. C. The same UMAP projection plot as (A), with detailed cell type information labeled. D. The correlations between cattle cell clusters and human cell clusters.

Fig. S5

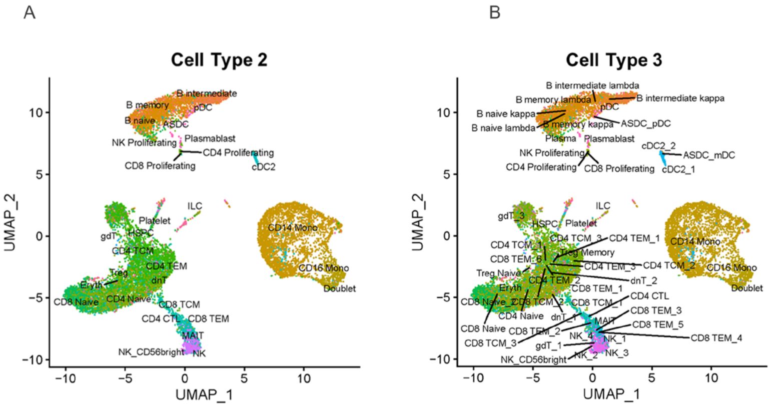

Figure S5. Comparative analyses between cattle and human PBMC under two resolutions using Azimuth. UMAP projection plot showing major cell type assignments for 13,601 individual cell transcriptomes from our cattle control sample and one human control PBMC sample in (A) medium and (B) high resolutions.

Fig. S6

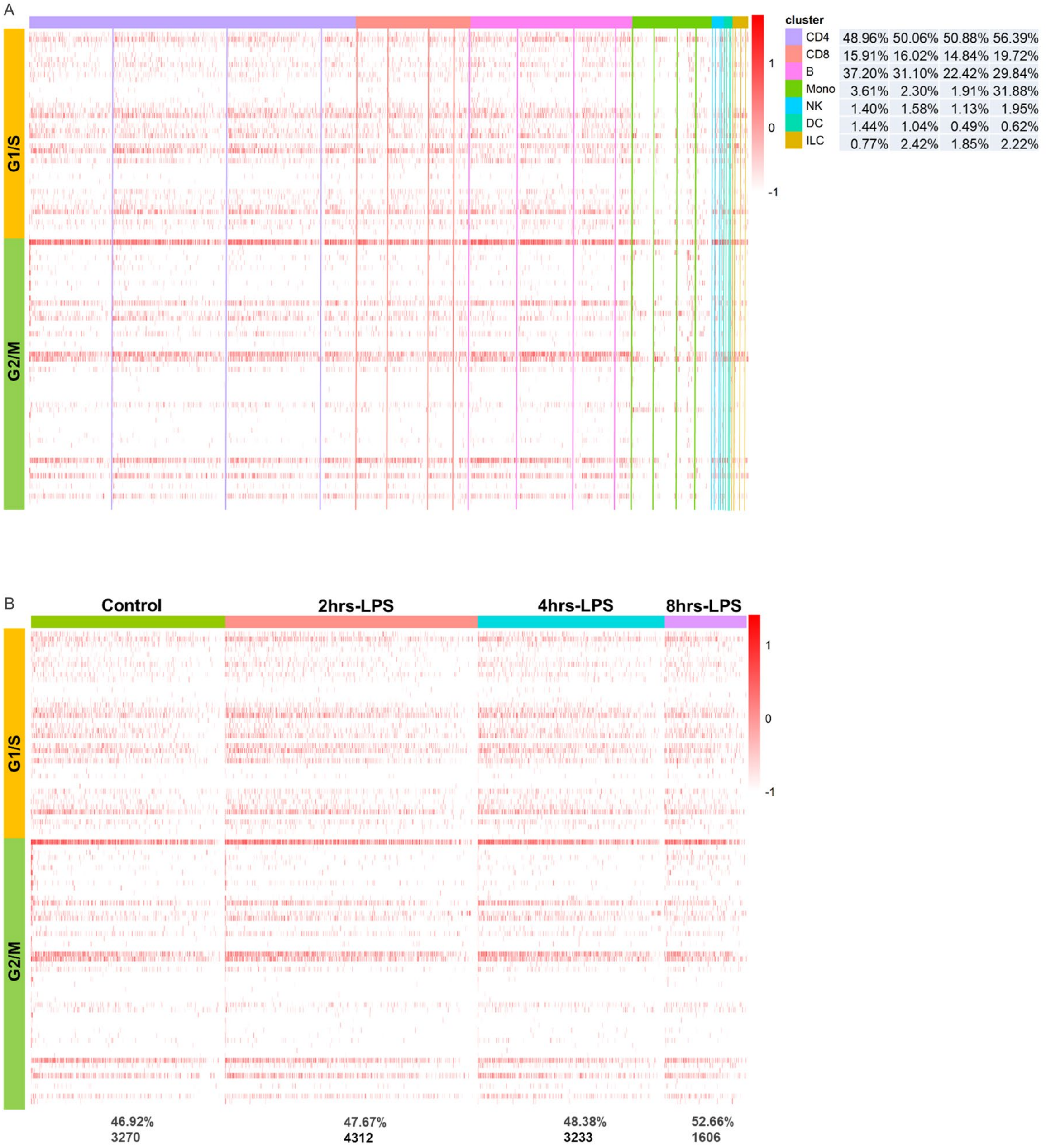

Figure S6. Cell cycle analysis for cattle PBMC. A. Heatmap showing expression levels of cell-cycle-related genes binned by seven cell types and then by the four treatments. Cells were ordered according to average expression level of cell-cycle-related genes within each cell. The color key from white to red indicated expression levels from low to high. The cell cycle index and cell count of each cell type across time series (Control, 2hrs-LPS, 4hrs-LPS and 8hrs-LPS) were shown at the right.

Fig. S7

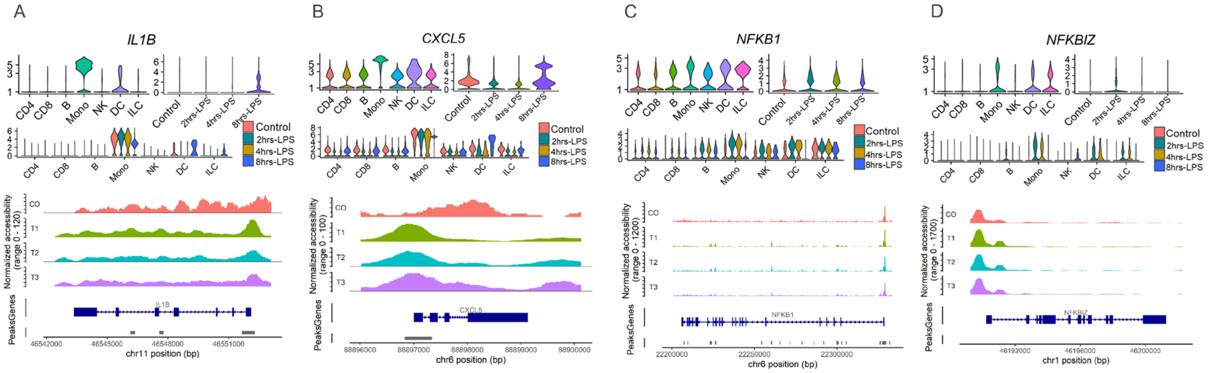

Figure S7. Gene expression of innate immunity genes and transcription factors during lipopolysaccharide treatments in cattle PBMC. Gene expressions and promoter chromatin accessibility of *IL1B* (A), *CXCL5* (B), *NFKB1* (C), and *NFKBIZ* (D) in four PBMC samples across different cell types and treatment time points.

Fig. S8

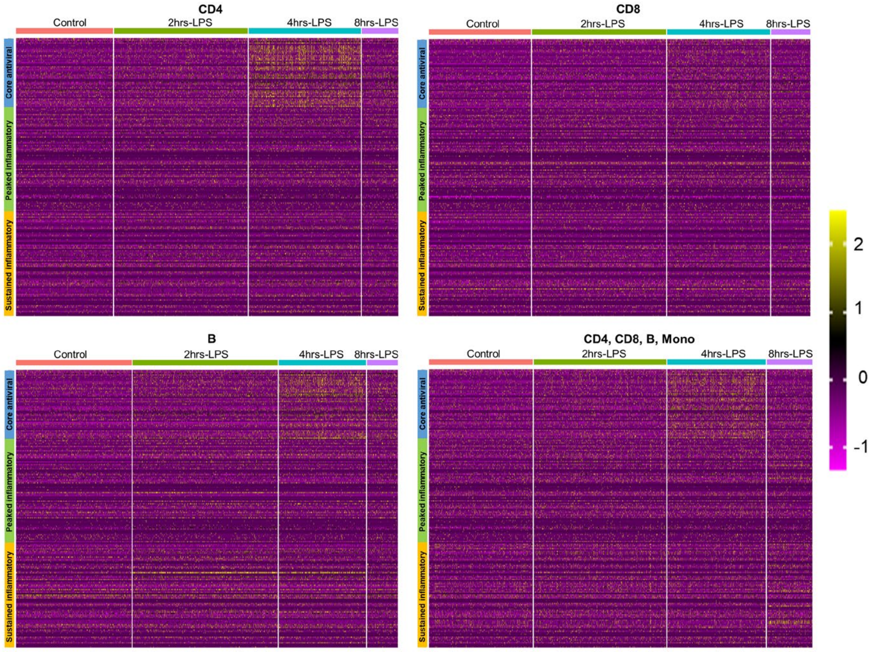

Figure S8. Heatmap showing scaled expression levels of three gene modules (core antiviral, peaked inflammatory, and sustained inflammatory) in CD4 cells, CD8 cells, B cells, separately and jointly with monocytes.
